# Genome Sequence of *Corynebacterium ulcerans* Strain 210932

**DOI:** 10.1128/genomeA.01233-14

**Published:** 2014-11-26

**Authors:** Marcus Vinicius Canário Viana, Leandro de Jesus Benevides, Diego Cesar Batista Mariano, Flávia de Souza Rocha, Priscilla Carolinne Bagano Vilas Boas, Edson Luiz Folador, Felipe Luiz Pereira, Fernanda Alves Dorella, Carlos Augusto Gomes Leal, Alex Fiorini de Carvalho, Artur Silva, Siomar de Castro Soares, Henrique Cesar Pereira Figueiredo, Vasco Azevedo, Luis Carlos Guimarães

**Affiliations:** a Department of General Biology, Federal University of Minas Gerais, Belo Horizonte, Minas Gerais, Brazil; b AQUACEN, National Reference Laboratory for Aquatic Animal Diseases, Ministry of Fisheries and Aquaculture, Federal University of Minas Gerais, Belo Horizonte, Minas Gerais, Brazil; c Department of Genetics, Federal University of Pará, Belém, Pará, Brazil

## Abstract

In this work, we present the complete genome sequence of *Corynebacterium ulcerans* strain 210932, isolated from a human. The species is an emergent pathogen that infects a variety of wild and domesticated animals and humans. It is associated with a growing number of cases of a diphtheria-like disease around the world.

## GENOME ANNOUNCEMENT

*Corynebacterium ulcerans* is a toxogenic zoonotic agent and Gram-positive bacterium that belongs to the *Actinobacteria* class, which includes the genera *Corynebacterium*, *Mycobacterium*, *Nocardia*, and *Rodococcus* and is referred to as a CMNR group. Studies using the 16S rRNA gene showed that *Corynebacterium pseudotuberculosis* and *Corynebacterium diphtheriae* are closely related to *C. ulcerans*. The species is facultative anaerobic, non–spore forming, nonmotile, catalase positive, and nitrate and oxidase negative. It differs from other species of the genus by fermentation of glycogen and starch (1).

The species can infect a variety of wild and domesticated animals and humans (2). It causes bovine mastitis and other infections in cats, dogs, monkeys, squirrels, otters, orcas, camels, lions, pigs, and goats. In humans, it causes diphtheria-like disease, pharyngitis, sinusitis, tonsillitis, pulmonary nodules, and skin ulcers (3). Contaminations in humans have been associated with raw milk and derivatives and contact with cattle and infected domestic pets (4). *C. ulcerans* is considered an emergent pathogen because the number of cases of infection in humans has been constantly increasing in the last two decades in the United States, Brazil, Western Europe, and Japan (5).

This species has a varied set of virulence factors, including *diphtheriae*-like toxin, phospholipase D, neuraminidase H, endoglycosidase EndoE, and a novel type of ribosome-binding protein with structural similarity to Shiga-like toxins. The sequencing of more *C. ulcerans* genomes, both toxigenic and non-toxigenic, will help in the identification of distinctive features of strains from human and animal sources, as well as in describing the zoonotic transmission in more detail (6). In addition, the data generated by newly sequenced genomes is helpful in identifying antibiotic and vaccine targets by comparative analysis (7). To date, only three complete genomes of *C. ulcerans* and two drafts have been deposited in the NCBI database.

Herein, we present the complete genome sequence of *Corynebacterium ulcerans* strain 210932, isolated from a human. Its genome sequencing was performed by the Ion Personal Genome Machine (PGM) System, using a fragment library. A total of 1,606,464 genomic reads were filtered by quality using the software FastQC (http://www.bioinformatics.babraham.ac.uk/projects/fastqc), and *de novo* assembling was done using Mira software version 3.9.18. The assembling step generated 12 contigs with a mean coverage of 129.23× and an *N*_50_ of 487,508. The contigs were scaffolded using the *C. ulcerans* strain 0102 as reference. The gaps were closed using CONTIGuator software (http://contiguator.sourceforge.net/) via the web tool SIMBA (SImple Manager for Bacterial Assemblies) (http://lgcm.icb.ufmg.br/simba/). CLC Workbench version 7 was used for manual curation of homopolymers, generating a final assembled genome with 2,484,335 bp.

An automatic annotation was done by RAST (http://rast.nmpdr.org/), followed by manual curation using Artemis software (http://www.sanger.ac.uk/resources/software/artemis/) and the Uniprot database (http://www.uniprot.org/). The genome has 2,282 coding sequences (from which 654, or 28.65%, were annotated as “hypothetical proteins”), 12 rRNAs, 51 tRNAs, and a G+C content of 53.32%.

### Nucleotide sequence accession number.

This whole-genome shotgun project has been deposited in GenBank under the accession number CP009500.
